# Multimorbidity and the indirect cost of productivity loss from health-related work absenteeism in Belgium

**DOI:** 10.1093/eurpub/ckaf063

**Published:** 2025-10-08

**Authors:** Phuong Bich Tran, Finaba Berete, Bart De Clercq, Johan Van der Heyden, Josefien van Olmen, Lander Willem

**Affiliations:** Department of Family Medicine and Population Health, University of Antwerp, Antwerp, Belgium; Department of Epidemiology and Public Health, Sciensano, Brussels, Belgium; Nuffield Department of Primary Care Health Sciences, University of Oxford, Oxford, United Kingdom; Department of Epidemiology and Public Health, Sciensano, Brussels, Belgium; Department of Research & Development, Mensura Group, Brussels, Belgium; Department of Epidemiology and Public Health, Sciensano, Brussels, Belgium; Department of Family Medicine and Population Health, University of Antwerp, Antwerp, Belgium; Centre for Research in Health System Performance (CRiHSP), Yong Loo Lin School of Medicine, National University of Singapore, Singapore; Department of Family Medicine (DFM), National University Health System, Singapore; Department of Family Medicine and Population Health, University of Antwerp, Antwerp, Belgium

## Abstract

This cross-sectional observational study aims to estimate the number of days absent from work due to health-related problems among employed individuals with multimorbidity and to quantify the lost productivity value from these absences. Data were obtained from the Belgian Health Interview Survey 2018, comprising employed individuals aged 15-64 (N = 4096). We examined 12 chronic conditions and 57 dyads. The Human Capital Approach was used by multiplying the reported number of days absent by the average wage per person per day, utilizing stratified gross wages from the Belgian Statistical Office. Approximately one-third of the study population reported multimorbidity. For individuals with zero to four+ chronic conditions, mean days of absence were 5.5 (95% CI: 2.3-8.8), 6.8 (95% CI: 2.9-10.7), 14.8 (95% CI: 10-19.6), 24 (95% CI: 17.8-30.2), and 36.2 (95% CI: 30.4-42), respectively. Depression (€3089; 95% CI: 2129-4049), diabetes (€2315; 95% CI: 962-3668), arthropathies (€1972; 95% CI: 1101-2844), and cancer (€1848; 95% CI: 598-3099), as standalone conditions, were associated with the greatest productivity losses. The effects were amplified up to seven times with the co-occurrence of multiple chronic conditions. We estimated 34.2 million days absent or €7.5 billion in lost productivity due to health-related work absenteeism among working-age employed individuals with multimorbidity in 2018. At the population level, the coexistence of two musculoskeletal disorders was linked to the highest aggregated productivity loss. At the individual level, the coexistence of a mental health condition and a somatic condition was associated with the highest average productivity loss per capita. The indirect cost due to health-related absence from work for individuals with multimorbidity in Belgium is high, and in many cases, exceeds the direct cost of treatment.

## Introduction

Health-related productivity loss is an important societal concept, encapsulating the economic impact of health issues on an individual's labor contribution [1]. It refers to the reduction in productivity due to health problems, measured by time away from work (absenteeism) and reduced productivity while at work (presenteeism) [2]. Multimorbidity affects both the young and the elderly [3] and can significantly increase absenteeism and presenteeism, hence reducing work performance compared to individual chronic conditions alone [4]. Furthermore, it is associated with increased healthcare utilization compared to single chronic conditions [5]. Exacerbated treatment and frequent healthcare visits [6] can, in turn, result in extensive time away from work. Therefore, it is paramount to consider multimorbidity in addition to standalone chronic conditions when evaluating productivity loss.

Belgium, like other high-income nations, grapples with challenges in sustaining its social security system due to an aging population [7]. Complicating this is the increasing prevalence of multimorbidity [8], affecting already nearly half of the population aged 15 and over [9], surpassing global (37.2%; 95% CI: 34.9–39.4) and regional prevalence rates (39.2%; 95% CI: 33.2–45.2) [10], though prevalence rates vary greatly depending on definitions and data sources. Multimorbidity not only strains the healthcare system, but also raises concerns about its potential long-term impact on the economy [11].

Most multimorbidity costing studies focused on direct costs (e.g. healthcare services and medication) [12]. Few studies accounted for indirect costs, such as the productivity losses resulting from health-related work absenteeism [12]. Both in high-income (Australia and Japan) and middle-income countries (Indonesia and China), multimorbidity has been linked to a substantial increase in average count of days absent among employed individuals, as well as a decreased likelihood of being employed despite being part of the labor force [13–15], yet specifics about disease combinations remain understudied. This lack of evidence hinders a comprehensive evaluation of how specific combinations are associated with productivity loss, specifically in terms of absenteeism [4].

The aim of this study was to quantify the economic impact of productivity loss among working individuals with multimorbidity, by estimating their number of days absent from work due to health-related problems.

## Methods

The study is a prevalence-based cost analysis and reported in line with the Strengthening the Reporting of Observational Studies in Epidemiology (STROBE) Statement [16] (Supplementary File S1).

### Data source

The Belgian Health Interview Survey (BHIS) is a repeated cross-sectional survey conducted every 4 to 5 years. It collects comprehensive health-related data from a representative sample of the Belgian population. Further details on sampling methods and survey design have been published elsewhere [17]. This analysis utilizes data from the BHIS conducted in 2018 and to a smaller extent, patient-level healthcare cost data estimated as part of a separate study using the administrative claim database 2017-2020 hosted by the Intermutualistic Agency [18].

### Population

Participants aged 15-64 who were employed (i.e. full-time employee, part-time employee, or self-employed—e.g. entrepreneurs) were included (N = 4096). As such, individuals in invalidity (e.g. severe health conditions or long-term disabilities) were not part of this analysis.

### Chronic conditions

The BHIS 2018 covered 38 chronic conditions (Table 1) and we used respondents' answers to the question, “During the past 12 months, have you had any of the following diseases or conditions?”. We consolidated these conditions into 25 groups, aiming to capture shared/similar clinical symptomatology among conditions. For example, we categorized conditions like epilepsy and chronic migraine under “episodic and paroxysmal disorders” due to their common characteristics of sudden and recurring occurrences. Our mapping of surveyed conditions was based on the work of Van Wilder et al. [9] and the International Statistical Classification of Diseases and Related Health Problems 10th Revision (ICD-10) [19].

**Table 1.** Classification of BHIS chronic conditions based on ICD-10 codes.

BHIS 2018 (38 chronic diseases)^a^	ICD-10 (25 chronic diseases)^b^
Allergy	Allergy (T78.4, J30.1)

Cancer	Cancer (C00-D49)

Chronic fatigue	Chronic fatigue (R53)

Chronic skin disease	Chronic skin disease (L80-L9)

Cirrhosis of the liver/liver dysfunction	Cirrhosis of the liver (K70-K77)

Disorder of the larger or the small bowel	Bowel disorder (K50-K52, K57, K58)

Gallstones of inflammation of the gallbladder	Gallstones (K80-K87)

Osteoporosis	Osteoporosis (M81, S70-S79)

Serious disease of the kidney	Kidney disease (N18, N19)

Serious gloom or depression	Depression (F33, F40, F41)

Stomach ulcer	Stomach ulcer (K25)

Stroke (or consequences)	Stroke (G45, I60-I69)

Thyroid problems	Thyroid problems (E00-E07)

Hypertension	Hypertension (I10-I15)

High cholesterol level in blood	High cholesterol (E78)

Low back disorder Neck disorder	Dorsopathies (M40-M54, M60-M63, M65-M68, M70-M79)

Rheumatoid arthritis Osteoarthritis/arthrosis	Arthropathies (M05.9, M13.0, M13.9, M15-M19)

Asthma Chronic bronchitis/COPD/emphysema	Respiratory disease (J40-J47)

Diabetes	Diabetes (E10-E14)

Epilepsy Severe headache (e.g. migraine)	Paroxysmal disorders (G40, G43, G44)

Macular degeneration Glaucoma Cataract Diabetic retinopathy	Eye disease (H35, H40-H42, H25, H26, H28)

Myocardial infarction Coronary heart disease Serious heart disease Narrowing of blood vessels in belly or legs	Cardiovascular disease (I20-I25, I48, I70-I79)

Urinary incontinence Stones in the kidney Chronic cystitis Prostate problems	Genitourinary problems (N03, N11, N18, N20-N23, N25-N29, N30-N39, N40-N51)

Parkinson‘s disease	Parkinson‘s disease (G20)
Hip fracture	Hip fracture (S70-S79)

^a^ **BHIS:** Belgian Health Interview Survey.

^b^ **ICD-10:** International Statistical Classification of Diseases and Related Health Problems 10th Revision.

### Multimorbidity measures

We defined multimorbidity as the presence of two or more ICD-10 based chronic conditions [3] as listed in Table 1.

### Absenteeism

We assessed the number of days absent using the question, “How many days in the last 12 months have you been absent from work due to a health problem?”. This question pertained to general health issues, including, but not specific to chronic conditions or multimorbidity. Furthermore, since the question only referred to the past 12 months, the influence of certain episodic conditions on our list (e.g. chronic fatigue, epilepsy, chronic migraine) on absenteeism may not have been captured within that time period.

### Missing data

Missingness for the variables used in the analysis was very limited. This is mainly due to the fact that these relevant survey questions were asked in the face-to-face interview. As such, interviewers posed questions to the respondents using a data entry program, in which relevant jumps were pre-programmed. For the majority of variables (relating to questions on chronic conditions and absence of work) missingness was <0.1%. For educational attainment, this was 1.8%. For income, missingness was higher since the survey also allowed respondents to provide income categories rather than a numerical value. Nevertheless, information on income was still available for almost 90% of the respondents. It was therefore decided that no imputation method would be performed and that a complete case analysis was acceptable for the statistical modelling. Moreover, another study utilizing the same dataset and similar variables compared the sociodemographic characteristics of the full and complete case samples and concluded that there were no particular differences [20].

### Cost perspectives

The study employed the Human Capital Approach to link an individual's productivity value to wages, which is essential for understanding societal costs [21]. To estimate the monetary value of lost productivity from health-related absenteeism (LPVA), we multiplied reported days absent by the average wage per person per day, utilizing gross wage data for the year 2018, from Belgium's National Statistical Office (Statbel) [22]. Average gross wages were gender-, region-, and seniority-specific. For years of seniority, certain cut-offs in the Statbel database were collapsed to fit with our age categories (see Supplementary File S2).

LPVA was expressed as 2018 Euro. Considering a standard year of 52 weeks with 5 working days per week and deducting 34 holidays, a full-time worker can work a maximum of 226 days in a year. To maintain accuracy, responses indicating more than 226 days of absence were capped at 226 [20].

In addition to individual burdens, we assessed population burden by multiplying average lost productivity per person per condition/combination by the prevalence rate of that condition/combination within the population under consideration.

### Analysis

We used generalized linear mixed models with quasi-Poisson distribution and log-link function to explore the relationships between chronic conditions, days absent, and lost productivity value. We accounted for the BHIS survey design and sampling weights in the models to ensure that the results were representative of the population from which the sample was drawn. Confidence intervals were calculated using the profile likelihood method. Statistical analyses and visualization were conducted in R, using RStudio version 2023.03.0 [23].

Further model specifications are detailed in Supplementary File S3. We developed two mixed models to individually assess the link between work absenteeism with single chronic conditions and dyads, respectively. Variable selection employed forward and backward stepwise regression analysis and priori knowledge with an initial 25 chronic conditions in Table 1 and seven covariates of interest (i.e. age, household education level, household income, healthcare costs, count of conditions, health-related quality of life (HRQoL) scores, and body mass index). Variables were added/removed based on p-values, with the Akaike Information Criterion (AIC) setting the threshold for the overall number of variables in the final model. *The single condition model* included 12 chronic conditions (i.e. allergy, arthropathies, cancer, cardiovascular disease, depression, diabetes, dorsopathies, chronic fatigue, genitourinary problems, hypertension, paroxysmal disorders, and stomach ulcer) and six determinants of illness-related work absences (i.e. age, household education level, healthcare costs, count of conditions, HRQoL scores, and body mass index). *The dyad model* complements the single condition model with an additional 20 interaction terms (Supplementary File S4). Predictions covered days absent and lost productivity value due to health-related work absenteeism for 57 observed dyads based on 12 chronic conditions.

## Results

### Study population characteristics

BHIS participants aged 15-64 accounted for 64% of the 2018 sample and around three-quarters were employed. Of the employed individuals aged 15-64 (N = 4096), 17% reported two chronic conditions, 9% reported three, and 10% reported four or more (Supplementary Table S5). Multimorbidity was reported by 36% (95% CI: 33.9-38.2) of the study population.

Except for the pairwise comparison between the sub-groups with 0 and 1 chronic condition, work absenteeism due to health problems was consistently higher among those with a higher count of chronic conditions in all other comparisons (*P*-value = .002 or less). The reported average days of absence were 5.5 (95% CI: 2.3-8.8) and 6.8 (95% CI: 2.9-10.7) for those without and with one chronic condition, respectively. The reported average days of absence were 14.8 (95% CI: 10-19.6) for those with two conditions, 24 (95% CI: 17.8-30.2) for those with three, and 36.2 (95% CI: 30.4-42) for those with four or more.

### Average days absent and LPVA associated with single chronic conditions

The prevalence, predicted average days absent, and LPVA per person per year for the included single chronic conditions and dyads are presented in Supplementary Files S6 and S7.

**Figure 1.**
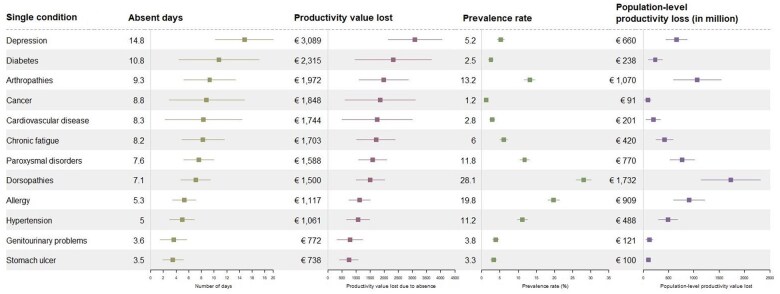
Days absent, LPVA (in 2018 Euro), prevalence, and population-level LPVA associated with single conditions. *Square object:* mean; *line:* confidence interval.

Among the 12 included single chronic conditions, which prevalence rates ranged from 1.2% to 28.1%, the average number of days absent due to health-related problems ranged from 3.5 to 14.8 (Fig. 1).

The chronic conditions associated with the highest number of days absent per person among the included conditions were depression, with an average of 14.8 days (95% CI: 10.2-19.5) or €3089 (95% CI: 2129-4049) in LPVA; diabetes [10.8 days (95% CI: 4.4-17.1) or €2315 (95% CI: 962-3668)]; arthropathies [9.3 days (95% CI: 5.2-13.4) or €1972 (95% CI: 1101-2844)]; and cancer [8.8 days (95% CI: 2.9-14.8) or €1848 (95% CI: 598-3099)]. The chronic conditions associated with the lowest absenteeism were stomach ulcer [3.5 days (95% CI: 1.9-5.1) or €738 (95% CI: 412-1064)] and genitourinary problems [3.6 days (95% CI: 1.5-5.7) or €772 (95% CI: 319-1224)]. The most prevalent chronic conditions were dorsopathies [7.1 days (95% CI: 4.7-9.4) or €1500 (95%CI: 994-2006)], allergy [5.3 days (95%CI: 3.5-7.1) or €1117 (95%CI: 739-1494)], and arthropathies [9.3 days (95%CI: 5.2-13.4) or €1972 (95%CI: 1101-2844)].

At the population level, combining individual burden and prevalence, dorsopathies (€1.7 billion), arthropathies (€1.1 billion), and allergies (€0.9 billion), as individual conditions, were associated with the highest average total days absent and LPVA. This was largely attributed to the high prevalence rates of these conditions.

### Average days absent and LPVA associated with dyads

**Figure 2.**
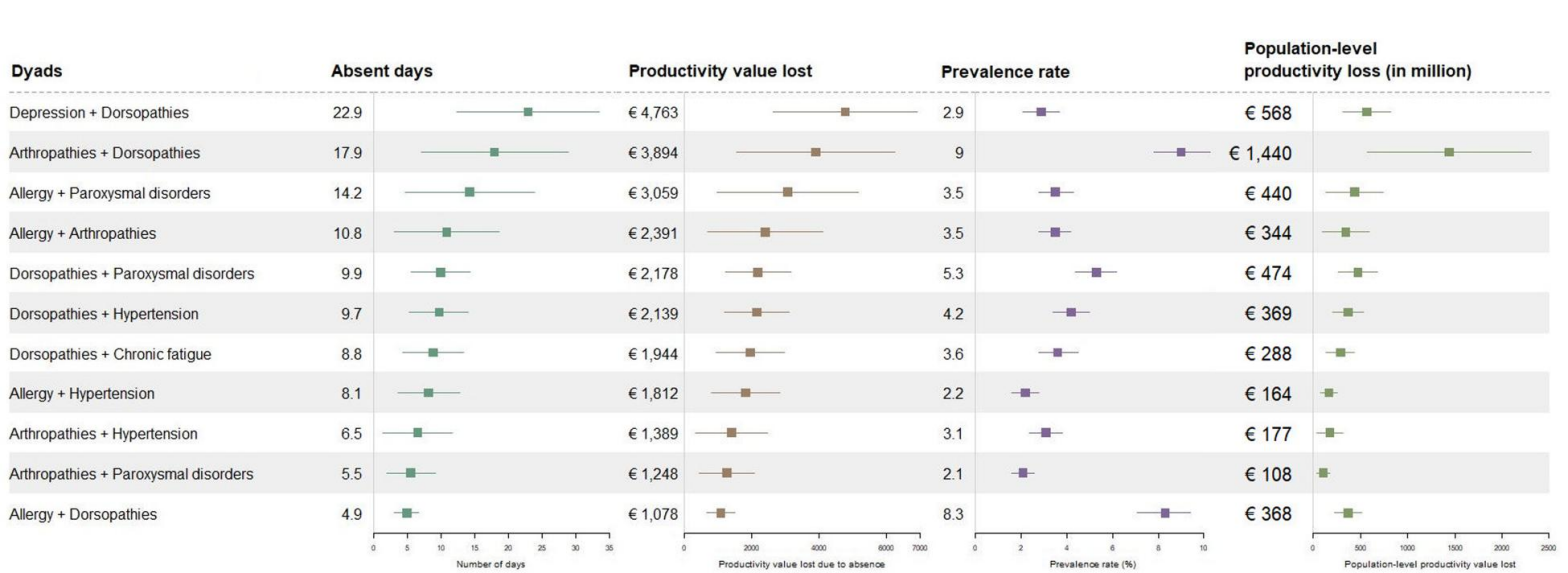
Days absent, LPVA (in 2018 Euro), prevalence, and population-level LPVA associated with dyads.

Among the most prevalent dyads (2.1% to 9.0%) of the 57 dyads considered, individuals on average had 4.9 to 22.9 days absent from work due to health-related problems (Fig. 2).

Depression + dorsopathies were linked with the highest average absenteeism per person among the included combinations, with 22.9 days (95% CI: 12.4-33.4), or €4763 (95% CI: 2618-6907) in LPVA. Allergy + dorsopathies were associated with the lowest average absenteeism per person, with 4.9 days (95% CI: 3-6.7), or €1078 (95% CI: 665-1491). The most prevalent dyad, arthropathies + dorsopathies, was associated with an average absence of 17.9 days (95% CI: 7.1-28.8), or €3894 (95% CI: 1541-6247).

The dyads associated with the highest average absenteeism had wider confidence intervals, which made it difficult to compare them to other dyads. However, dyads with the most and fewest days absent (e.g. depression + dorsopathies *vs* allergy + dorsopathies) could be compared as their confidence intervals did not overlap. In this case, having depression, as opposed to allergies, among individuals with dorsopathies was associated with a notable increase in the number of days absent due to health-related problems. Having allergy + dorsopathies together was also associated with reduced health-related work absence, compared to when these conditions were experienced separately (n = 307; *P* = .002).

Notably, having two musculoskeletal disorders (arthropathies + dorsopathies) was associated with the second highest average absence per person of all considered dyads, and higher than the summed average days absent associated with the individual conditions, though this is not statistically significant.

At the population level, arthropathies + dorsopathies (€1.4 billion), depression + dorsopathies (€0.6 billion), and dorsopathies + paroxysmal disorders (€0.5 billion), were associated with the highest combined total days absent and LPVA, largely due to their extensive prevalence rates.

### Total absenteeism and LPVA

**Figure 3.**
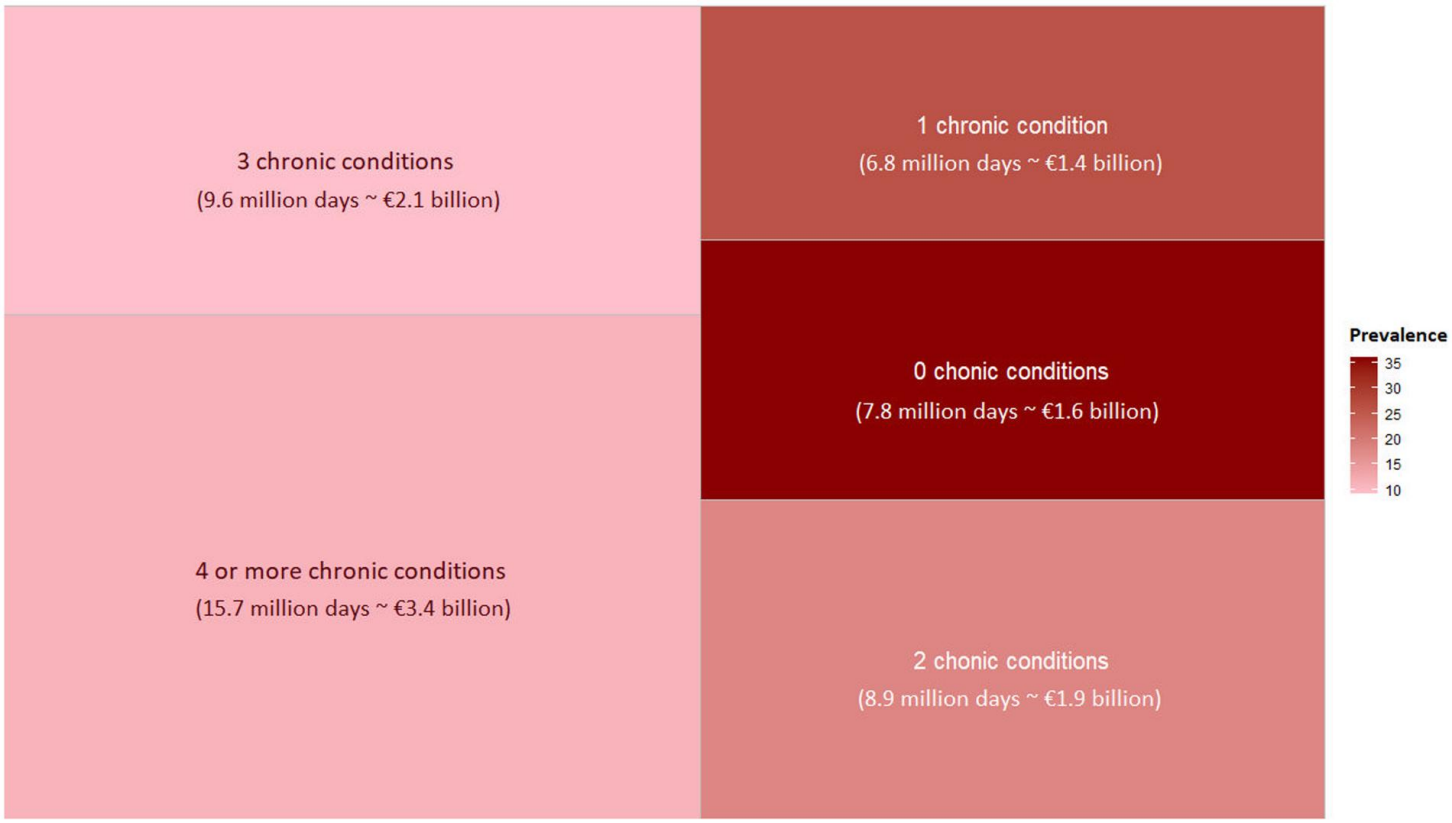
Aggregated absenteeism and LPVA (in 2018 Euro) by number of chronic conditions.

When extrapolating to the entire employed Belgian population aged 15-64 (including the few cases whose multimorbidity status or morbidity count could not be identified), we estimated a total of 49.3 million days absent (95% CI: 42.7-56.0) or €10.6 billion (95% CI: 9.1-12.0) in LPVA, which was roughly 2.3% of Belgium’s gross domestic product (GDP) in 2018 (Fig. 3).

Among this, 34.2 million days absent (95% CI: 28.2-40.2) or €7.5 billion (95% CI: 6.2-8.8) in LPVA were estimated for working-age employed individuals with multimorbidity, which was around 70% of all health-related work absenteeism productivity loss and 1.6% of Belgium’s GDP.

Among those whose morbidity count was known, we estimated 6.8 million days absent (€1.4 billion) in people with one chronic condition. For people with two, three, and four+ chronic conditions, we estimated a total of 8.9 million days absent (€1.9 billion), 9.6 million days (€2.1 billion), and 15.7 million days (€3.4 billion), respectively. The extrapolated total numbers of days absent were greater in sub-groups featuring a higher count of co-existing conditions. This variation depends on the prevalence, morbidity patterns, as well as the number of days absent per person per morbidity combination. These estimations were based on the reported total number of days absent for each mutually exclusive group in our study population (i.e. based on the total number of days absent for all individuals with one, two, three, and four+ co-existing conditions, respectively), adjusting for sampling weights.

## Discussion

This study examines the link between multimorbidity and productivity loss, focusing on health-related work absenteeism in employed individuals aged 15-64 in Belgium.

### Contextualizing the relationship between multimorbidity and productivity loss

Approximately one-third of the study population reported multimorbidity. Health-related work absenteeism was three to seven times higher than in individuals with no chronic conditions, and two to five times higher than individuals with one condition. In the United States, individuals with multimorbidity had a 5.9-fold increase in lost workdays versus those without chronic conditions and a 2.5-fold increase versus those with one condition [24]. However, comparing findings across settings and populations is challenging due to variations in regulations, policies, and work cultures. Multiple factors influence absenteeism, including demographic characteristics, education, income, occupational class, professional grade, social support, work environment, shift work, and illness identity [25, 26]. Clinical factors like duration, severity, symptom exacerbation frequency, and healthcare-seeking patterns translate into differences in work absence between morbidities.

### The relationship between specific conditions/combinations and loss of productivity

Depression, diabetes, arthropathies, and cancer as standalone conditions were linked to the highest productivity loss among the chronic conditions studied. In Australia, the United Kingdom, France, and Finland, musculoskeletal disorders and mental/neurological conditions were also found to have the highest impact [27, 28]. In the Netherlands, musculoskeletal disorders, mental health conditions, and cancer, as individual conditions, substantially increased sick leave [29]. Diabetes ranked second in its association with productivity loss in our analysis, aligning with other studies highlighting its substantial impact on work loss [30, 31]. Uncontrolled diabetes, indicated by poor glycemic control, has been linked to high absenteeism rates, with the indirect costs primarily stemming from disability due to the condition’s complications rather than the condition itself [32].

Among the analyzed dyads, arthropathies + dorsopathies resulted in the highest overall productivity loss at the population level. With a prevalence of 9%, the estimated total lost productivity was approximately €1.4 billion, equivalent to about 0.3% of Belgium's GDP. This combination also showed the second-highest average absence per individual among dyads, exceeding the combined average absence days related to the individual conditions. Although not statistically significant, this suggests that the presence of both musculoskeletal disorders might amplify the impact on work absenteeism, potentially through decreased functionality or increased pain intensity beyond that experienced with each disorder independently.

Depression + dorsopathies were associated with the highest average productivity loss per person. Previous studies linked HRQoL to absenteeism [33] and the HRQoL score for depression + dorsopathies [0.49 (95% CI: 0.48-0.51)] was one of the lowest in Belgium [9]. Furthermore, a super-additive effect has been observed where the presence of a mental disorder amplifies the number of days absent in individuals with a physical disorder. Emotional stress is proposed to heighten pain perception and contribute to a motivational deficit, prolonging work absence [33]. Musculoskeletal disorders and mental health issues, including stress, depression, and anxiety, rank among significant occupational health problems in Europe [34]. This underscores the need for integrated prevention strategies addressing both these health issues simultaneously.

### Interaction effects of co-occurring conditions on productivity loss

Incorporating interaction effects is important to understand particularly detrimental morbidity combinations [27]. Some combinations were linked to an exacerbated effect of individual conditions. For instance, having two musculoskeletal conditions may increase associated productivity loss compared to having just one, but having allergy and a musculoskeletal condition may not lead to additional productivity loss. Note that these results partly depend on model specifications, morbidity inclusion, and prevalence. In our model, many multimorbidity interaction coefficients were statistically insignificant, suggesting that observed absence in most combinations did not significantly differ from the expected additive absence. The consistent trend of increasing productivity loss with higher morbidity counts suggests a partially additive effect [27]. Specifically, the average productivity loss associated with dyads was 2.7 times that of having no chronic conditions, and average productivity loss of triads was 3.5 times that of one condition. Having four+ conditions was associated with a lost productivity value 2.4 times that of dyads. While this study does not allow us to explain all multimorbidity patterns, it lays the groundwork for exploring the health profiles and sick leave needs of individuals with combinations exhibiting super- or sub-additive absenteeism. Factors like disease progressions, diminished HRQoL, frequent healthcare utilization, work environment, policies, social support systems, attitudes toward illness, and sickness identity could play a role in driving the super-additivity of absenteeism [26, 35].

### The direct cost versus indirect cost of multimorbidity

Many cost-of-illness studies are conducted from a payer perspective, focusing on direct costs [12]. However, incorporating indirect societal costs (i.e. opportunity costs) might alter the conclusions in economic evaluations [36]. Building on a prior study on the direct cost of multimorbidity in Belgium, we deem it important to observe patterns of indirect costs relative to their corresponding direct costs for specific morbidity combinations [18]. For the most prevalent dyad, arthropathies + dorsopathies, the average direct cost was €3044 per person per year, while the estimated average indirect cost was €3894. Depression + dorsopathies had an annual average direct cost of €2537, with the corresponding indirect cost nearly twice the direct cost, totaling €4763. These findings highlight that, in many cases, the indirect cost of multimorbidity to society exceeds its direct cost of treatment.

Cancer, diabetes, chronic fatigue, and genitourinary problems were observed with the highest medical costs in Belgium [18], placing a significant burden on the healthcare fund. On the other hand, depression, diabetes, arthropathies, and cancer had the greatest association with productivity loss from health-related work absence, posing a considerable strain on the economy and society. Individually, cancer and diabetes consistently appeared among the costliest chronic conditions in terms of both direct and indirect costs. The burden of multimorbidity creates a challenging situation by straining the healthcare budget and hindering taxpayers from working (i.e. long-duration absences and invalidity), leading to a vicious cycle of reduced production and increased use of public healthcare resources [11].

### Implications for future research, health system, and policy

In total, 34.2 million days absent or €7.5 billion in lost productivity value were estimated for individuals with multimorbidity, constituting around 70% of all health-related work absenteeism productivity loss and 1.6% of Belgium's GDP in 2018. Though limited to the 15-64 age group, this indicates a substantial societal loss. Despite the argument that short absences may not immediately translate into tangible costs [37], Belgian panel data suggest that a 1% increase in absenteeism rates corresponds to a 0.24% decline in firm output, impacting certain sectors more significantly (e.g. industrial firms) [38]. Additionally, chronic conditions persist in the long term and tangible costs are inevitable.

Our findings highlighted specific combinations that have a substantial association with work absence and productivity loss. Longitudinal datasets and further examination of causal relationships can support healthcare providers, policymakers, and employers to develop targeted interventions, care innovations, and support strategies for individuals affected by these combinations. We also identified morbidity combinations that were associated with the highest productivity loss on the individual and population level. In case causalities are established in further research, interventions may be considered differentially for these two groups. Corporate prevention strategies and periodical health examinations should be aimed at morbidity combinations with high prevalence and low-moderate individual productivity impact, in order to control disease progression, prevent complications and the onset of additional comorbidities [39]. For both groups, but particularly combinations with detrimental impacts on individuals’ productivity, tailored disease management programs, workplace accommodations, and strategies to improve overall health and well-being should be prioritized [39].

Further research is needed to understand the underlying mechanisms and factors driving the absenteeism rates observed in these combinations. Moreover, additional research could be undertaken to delve into presenteeism; i.e. exploring the patterns of reduced productivity at work due to illness—which has previously shown to be higher than the cost of absenteeism [40].

### Strengths and limitations

Our study addresses an important research gap on productivity loss due to multimorbidity. Beyond the number of conditions, we assessed the relationship per morbidity profile, examining 12 chronic conditions and 57 dyads. By focusing on indirect costs, this study complements existing research on the direct costs of multimorbidity and contributes to a societal perspective of its economic impact in Belgium. The findings are generally applicable to the specific study population under examination, owing to the rigorous sampling strategies employed in the BHIS.

This study had certain limitations. It does not establish causal inferences, emphasizing interpretation within the context of distinctions observed in absence/productivity loss across multimorbidity and non-multimorbidity profiles. The focus was solely on paid-work absences and did not include presenteeism, unpaid work, premature retirement, or mortality. The analysis was limited to an individual's productivity loss and did not account for effects on family members (e.g., caregiving responsibilities). Due to low prevalence, mortality, or inclusion bias, certain high-lethality conditions such as stroke were omitted; as a result, some values may be overestimated because of the masking effects of excluded severe conditions. The healthy volunteer effect led to the exclusion of less healthy individuals, for example cancer non-survivors. The study excluded those with severe health states or long-term disabilities who were outside of the workforce. Certain combinations had limited observations, yielding moderate certainty in estimates. The use of self-reports rather than clinical diagnoses has limitations. However, self-assessment provided insights into conditions and symptoms affecting workability, recognising that not all individuals seek healthcare for every condition. Absenteeism data were also based on self-reports, which may have introduced recall bias and inconsistencies. Future research could benefit from linking BHIS data with labor information from the Belgian Crossroads Bank of Social Security, fostering collaboration among relevant authorities and institutions. The work loss question pertained to the 12 months before the interview, which may not have properly captured the occurrence or impact of episodic conditions. Specific health reasons for the absences were not discernible and some cases may have been due to accidents or acute events rather than chronic conditions.

Our study evaluated the productivity loss from health-related work absence associated with multimorbidity, encompassing 12 chronic conditions and 57 dyads. Health-related work absenteeism, on average, increased substantially with multimorbidity compared to those with no chronic conditions or one chronic condition. With over a third of the working-age population reported with multimorbidity, our detailed insights on specific disease combinations allow us to understand the economic and societal burdens associated with multimorbidity and identify potential unmet needs.

## Supplementary Material

ckaf063_Supplementary_Data

## Data Availability

The data that support the findings of this study are available from Sciensano. However, restrictions apply to their availability, as they were used under license for the current study and are not publicly accessible. Data may be made available upon reasonable request and with permission from Sciensano.
