# Supplementary material for: Multimorbidity and the indirect cost of productivity loss from health-related work absenteeism in Belgium
Source: Eur J Public Health. 2025 Oct 8;35(6):1129–36. doi: 10.1093/eurpub/ckaf063 (PMC12707494; doi:10.1093/eurpub/ckaf063)
Supplement: ckaf063_Supplementary_Data [file ckaf063_supplementary_data.zip › ejph-2024-08-om-0514-File008.docx]

**Model equations:**

${log (days or indirect costs)}_{ij}$ = $\beta_{0_{days or indirect costs}}$ + $\sum_{m=1}^{12} \beta_{ijm}M_{ijm}$+ $\sum_{c=1}^{6} \beta_{ijc}C_{ijc}$ + ($u_{ij}$+ $\varepsilon_{i}$)

And:

${log (days or indirect costs)}_{ij}$ = $\beta_{0_{days or indirect costs}}$ + $\sum_{m=1}^{12} \beta_{ijm}M_{ijm}$+ $\sum_{d=1}^{20} \beta_{ijd}D_{ijd}$ + $\sum_{c=1}^{6} \beta_{ijc}C_{ijc}$ + ($u_{ij}$+ $\varepsilon_{i}$)

Where:

${log (days or indirect costs)}_{ij}$ the estimated log-transformed value on the absent day and indirect cost variable for individual $i$ from household $j$, respectively;

$\beta_{0_{days or indirect costs}}$ the fixed effects that represent the overall mean of ${log (days or indirect costs)}_{ij}$across all households $j$ for individuals $i$ of the reference category, respectively;

$M_{ijm}$ a vector representing 12 individual-level chronic morbidities and $\beta_{ijm}$ are their respective estimated fixed slope coefficients;

$D_{ijd}$ a vector representing interaction terms of dyads and $\beta_{ijd}$ are their respective estimated fixed slope coefficients;

$C_{ijc}$ a vector representing the six individual-level confounding factors and $\beta_{ijc}$ are their respective estimated fixed slope coefficients;

The random part between brackets includes: $u_{ij}$ and $\varepsilon_{i}$. The first allows the intercept to vary between households, accounting for the household specific deviations from the overall intercepts $\beta_{0_{days or indirect costs}}$. The second is the idiosyncratic error term, which accounts for the individual deviations in ${log (days or indirect costs)}_{ij}$from the household specific intercept.

Note that with only over 4000 observations distributed unevenly across regions—61% in Flanders, 9% in Brussels, and 29% in Wallonia—we did not incorporate region as random effects as this complicated the model, and insufficient data to support such granularity led to issues of model convergence and overfitting.
